# ABA–ethylene crosstalk accelerates persimmon fruit softening via induction of *DkNAC26* and *DkNAC28*

**DOI:** 10.1093/hr/uhag055

**Published:** 2026-02-28

**Authors:** Yaxiu Xu, Fan Yang, Huiru Song, Xinru Zhao, Hui Gao, Ningjing Sun, Xiaofen Liu, Xueren Yin, Yuduan Ding, Qinggang Zhu

**Affiliations:** College of Horticulture, Northwest A&F University, Yangling, Shaanxi 712100, China; College of Horticulture, Northwest A&F University, Yangling, Shaanxi 712100, China; College of Horticulture, Northwest A&F University, Yangling, Shaanxi 712100, China; College of Horticulture, Northwest A&F University, Yangling, Shaanxi 712100, China; College of Food Science and Technology, Northwest University, Xi’an 710069, China; College of Resources and Environment Sciences, Baoshan University, Baoshan, Yunnan 678000, China; School of Horticulture, Anhui Agricultural University, Hefei 230036, China; School of Horticulture, Anhui Agricultural University, Hefei 230036, China; College of Horticulture, Northwest A&F University, Yangling, Shaanxi 712100, China; College of Horticulture, Northwest A&F University, Yangling, Shaanxi 712100, China

## Abstract

Ethylene and abscisic acid (ABA) play crucial roles in the ripening and softening of persimmon fruit, and they can promote each other to accelerate the softening process. However, the underlying molecular mechanisms remain to be further elucidated. In this study, a transcription factor *NAM ATAF1/2, CUC26 (DkNAC26*) induced by ethylene was identified. It could increase the content of ABA in persimmon fruit by promoting the expression of ABA synthesis-related gene *DkNCED2 + 3′*, thereby reducing fruit firmness. On the other hand, ABA could induce the expression of transcription factor *DkNAC28*, which binds to the promoter region of the ethylene biosynthesis gene *DkACS1*, leading to an earlier ethylene burst and consequently accelerating fruit softening. This study elucidates the functional roles of two transcriptional activators, DkNAC26 and DkNAC28, in regulating the biosynthesis of ethylene and ABA and reveals a molecular mechanism through which these two hormones interact to promote fruit softening, providing a new perspective for hormone crosstalk that drives rapid softening in persimmon.

## Introduction

Persimmon is valued for distinctive flavor and texture, yet rapid postharvest ripening and softening compromise marketability. Fruit softening is a complex process involving pectin and hemicellulose depolymerization in the primary cell wall, changes in cell turgor, and altered tissue water status [1–3]. Pectin degradation reduces intercellular adhesion and is a major driver of softening [4]. Plant hormones are pivotal regulators of softening, with ethylene and abscisic acid (ABA) acting as key signals [5, 6].

Ethylene is the principal regulator of climacteric ripening and softening, whereas ABA is central in many nonclimacteric systems [5, 6]. In ethylene biosynthesis, amino cyclopropane carboxylic acid (ACC) is the direct precursor and ACC synthase (ACS) is rate limiting [7, 8]. Loss of function in *MdACS1* suppresses ethylene production in apple (*Malus domestica*) and impairs ripening [9, 10]. Exogenous ethylene accelerates softening in multiple species including apple, banana (*Musa* spp.), peach (*Prunus persica*), and kiwifruit (*Actinidia deliciosa*) [11–13]. ABA also modulates climacteric ripening; exogenous application promotes softening in several fruits [5, 14–17]. ABA biosynthesis starts with cleavage of a C40 carotenoid, with plastid-localized 9 *cis* epoxycarotenoid dioxygenase (NCED) acting as a key control point; manipulation of *NCED* genes alters ABA accumulation and ripening in tomato and peach，*DkNCED2 + 3′* involved in ABA synthesis were identified in Persimmon [18–20].

Ethylene and ABA jointly regulate ripening and softening. Ethylene-induced PpERF3 activates *PpNCED2* and *PpNCED3* to promote ABA biosynthesis and softening in peach [21]. ABA can induce endogenous ethylene, accelerating mango softening [22]. In tomato (*Solanum lycopersicum*), co-silencing ABA receptor genes (*SlRCAR9*, *SlRCAR12*, *SlRCAR11*, *SlRCAR13*) suppresses ethylene biosynthesis and signaling, delaying ripening and increasing firmness [23]. Blocking ABA signaling does not abolish ethylene-induced ripening but attenuates ABA promotion of ripening, suggesting ABA may act upstream of ethylene in some contexts [24]. SINAC1 modulates tomato ripening and softening via both ethylene and ABA pathways [23]. However, the mutual regulation between ethylene and ABA in persimmon (*Diospyros kaki*) remains poorly defined.

NAC transcription factors play a regulatory role in the ripening and softening processes of fruit. In kiwifruit, AcNAC1 and AcNAC2 activated *AcXTH1* and *AcXTH2* to promote ripening and softening [25]. In apple, MdNAC18.1 upregulated softening genes such as *PG* and ethylene biosynthetic genes including *ACS* [26]. Nitric oxide inhibited SlNAP2 reduces transcription of *SlCCEL2*, *SlPG2a*, and *SlPL8*, diminishing cell wall enzyme activities and delaying softening [27]. In persimmon, DkNAC1, DkNAC3, DkNAC5, and DkNAC6 are implicated in astringency removal [28], and DkNAC9 activates *DkEGase1* in concert with DkERF8 and DkERF16 to promote softening post-deastringency [29]. Additional NAC-mediated mechanisms controlling persimmon ripening and softening remain to be defined.

## Results

### Mutual promotion by ethylene and ABA accelerates persimmon fruit softening

**Figure 1:**
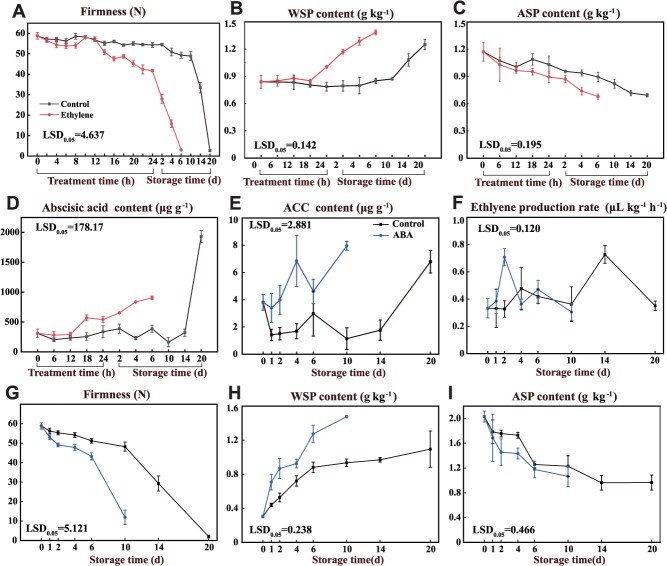
Ethylene and ABA enhanced softening of persimmon fruit. (A) Firmness, (B) soluble pectin, (C) protopectin, and (D) ABA content of persimmon fruit after ethylene treatment. (E) ACC content, (F) ethylene production, (G) firmness, (H) soluble pectin, and (I) protopectin content of persimmon fruit after ABA treatment. Values are the means (±SE) from three biological replicates. The LSD_0.05_ values refer to mean comparisons between treated and untreated samples at the same time point.

Exogenous ethylene treatment promoted fruit softening and increased ACC content and stimulated endogenous production in persimmon (Fig. S1, S2A, and  B). Ethylene treatment reduced fruit firmness and accelerated the conversion of protopectin to soluble pectin, thereby increasing the softening rate (Fig. 1A–C). To test whether ethylene influences ABA, we quantified ABA and found that ethylene increased endogenous ABA content (Fig. 1D). ABA could also accelerate the softening of persimmon fruit (Fig. S3). Meanwhile, exogenous ABA increased endogenous ABA and ACC content, advancing the ethylene peak (Figs 1E and F and Fig. S4). ABA treatment also reduced firmness and accelerated protopectin to soluble pectin conversion (Fig. 1G–I). Together, these data show reciprocal enhancement between ethylene and ABA that accelerates persimmon fruit softening.

### Transcriptome analysis identifies ethylene-induced transcription factors in persimmon fruit

**Figure 2:**
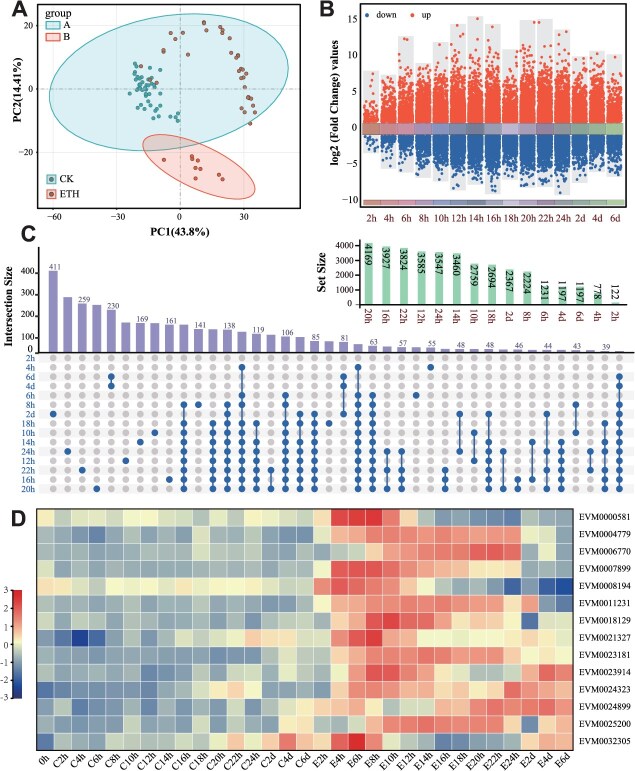
The transcriptomic analysis of persimmon fruit treated with ethylene and control. The persimmon fruit were treated with ethylene (E) and control (C) and then storage at 20°C. (A) PCA of transcriptome data from each time point of persimmon fruit during storage time. (B and C) The upset plots and the volcano plots show the number of DEGs at different sampling points and interaction set between two sampling points. (D) Heatmap of transcription factors with differential expression between control and ethylene-treated fruit from the RNA-seq data.

After exogenous ethylene treatment, ACC levels and endogenous ethylene production increased rapidly in persimmon fruit (Fig. S2A and B). Therefore, we performed intensive early-time sampling, collecting tissues every 2 h over the first 24 h of treatment. To better understand how ethylene regulates fruit softening, we performed transcriptome sequencing on ethylene-treated (E) and control (C) fruit. The transcriptome data were analyzed using principal component analysis (PCA) and correlation analyses of differentially expressed genes (DEGs). Based on these data, many DEGs responded rapidly to ethylene after treatment (Fig. 2A–C). To probe early ethylene signaling, DEGs were screened based on transcriptome fragments per kilobase of transcript per million mapped reads (FPKM) quantity. The screening criteria were |log2(FPKM_E/FPKM_C)| > 1 and FPKM_E > 20 on DEGs at 0–8 h. A total of 14 ethylene-induced transcription factors were identified (Fig. 2D). These transcription factors are candidate regulators mediating ethylene-triggered increases in endogenous ABA.

### DkNAC26 enhances the promoter activity of the ABA biosynthesis gene *DkNCED2 + 3’*

**Figure 3:**
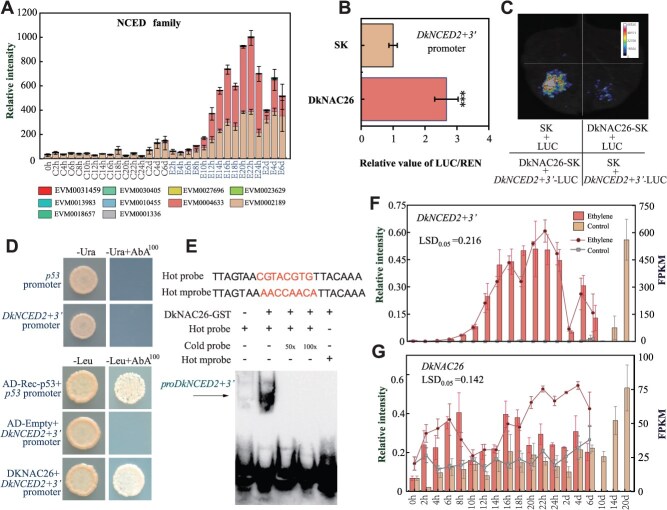
DkNAC26 enhances the promoter activity of *DkNCED2 + 3′* . (A) Analysis of FPKM values for the NCED gene families was presented. (B) Dual-luciferase assays were performed to detect the regulatory effects of DkNAC26 on the promoter of *DkNCED2 + 3′*. The LUC/REN fluorescence ratio obtained from the empty vector pGreenII 002962-SK (SK) plus promoter was set to 1. Values are presented as mean (±SE) of three biological replicate assays. (C) Firefly LCI in *N. benthamiana* leaves. (D) Y1H assay shows binding of DkNAC26 to the *DkNCED2 + 3′* promoter. The basal concentrations of aureobasidin A (AbA) used were 100 ng·ml^–1^. The empty vector with the *DkNCED2 + 3′*promoter served as negative controls and Rec-P53 and the P53 promoter were used as the positive controls. (E) EMSA showing that DkNAC26 binds to the NAC-binding of the *DkNCED2 + 3′* promoter. The hot probe was a biotin-labeled *DkNCED2 + 3′* promoter fragment (CGTA and CGTG), and the cold probe was an unlabeled competitive probe (50- and 100-fold that of the hot probe). A mutant hot probe comprised a hot probe sequence with eight mutated nucleotides. GST-tagged DkNAC26 was purified. The expression levels of *DkNCED2 + 3′* (F) and *DkNAC26* (G) were detected by RT-qPCR. The histogram represents the RT-qPCR data, and the line chart illustrates the FPKM values obtained from the transcriptome data. Values are means (±SE) from three biological replicates. The LSD_0.05_ values refer to mean comparisons between treated and untreated samples at the same time point.

During ABA biosynthesis, NCED directly influences ABA production (Fig. S5). Therefore, we analyzed the differential expression of NCED genes following ethylene treatment and observed that ethylene induced the expression of *DkNCED2 + 3′* (EVM0004633) (Fig. 3A). The regulatory effects of 14 differentially expressed transcription factors on *DkNCED2 + 3′* promoter were assessed using a dual-luciferase reporter assay system. The results demonstrated that DkNAC26 (EVM0024899) enhanced the activity of the *DkNCED2 + 3′* promoter with fold changes of 2.7 (Figs 3B and C, and S6). To further verify the interaction between DkNAC26 and the *DkNCED2 + 3′* promoter, a yeast one-hybrid assay was conducted. The results demonstrated that DkNAC26 bound to the *DkNCED2 + 3′* promoter (Fig. 3D). To further confirm this interaction, we purified the full-length DkNAC26 protein and performed an electrophoretic mobility shift assay (EMSA) with fragments of the biotin-labeled *DkNCED2 + 3′* promoter containing 2 NAC-binding sites (CGTA and CGTG) as the labeled probe. DkNAC26 bound directly to both NAC-binding sites (Fig. 3E). Real-time quantitative PCR (RT-qPCR) showed that the expression levels of both *DkNAC26* and *DkNCED2 + 3′* were elevated following ethylene treatment compared with the control (Fig. 3F and G). Collectively, these findings indicate that ethylene-induced *DkNAC26* enhances the expression of the ABA biosynthesis gene *DkNCED2 + 3′*, thereby increasing ABA content in persimmon fruit.

### RNA-seq analysis and identification of key transcription factors and structural genes involved in ethylene biosynthesis in response to ABA during persimmon fruit ripening

**Figure 4:**
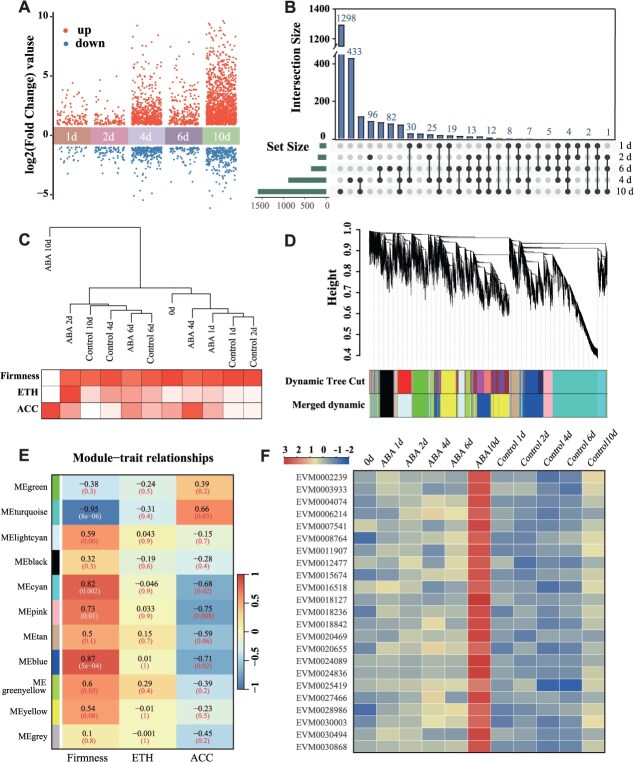
The transcriptomic analysis of persimmon fruit treated with ABA and control. The persimmon fruit were treated with ABA and control and then stored at 20°C. (A and B) The upset plots and the volcano plots show the number of DEGs at different sampling points and interaction set between two sampling points. (C) Hierarchical clustering analysis of samples based on transcriptomic profiles. (D) Gene-clustering tree and module division. (E) WGCNA analysis revealed clusters of coexpressed genes and metabolites associated with specific phenotypic traits. The table presents correlations between gene modules and traits, along with their corresponding *P*-values (shown in parentheses). Each row represents a distinct module, while each column corresponds to a physiological characteristic, including firmness, ethylene production, and ACC levels. The color gradient at the bottom left illustrates module-trait correlation from −1 (blue) to 1 (red). (F) Heatmap of transcription factors with differential expression between control and ABA-treated fruit from the RNA-seq data.

Transcriptome sequencing was performed on both ABA-treated and control fruit. Weighted gene coexpression network analysis (WGCNA) was performed using transcriptome data (incorporating 2430 DEGs) and phenotypic traits (fruit firmness, ACC content, and ethylene production). Genes with similar expression patterns were grouped into modules (Fig. 4A–E). Eleven distinct coexpression modules were identified. The MEturquoise module was prioritized for further analysis due to its strong negative correlation with fruit firmness (*r* = −0.95). Within MEturquoise, candidate transcription factors potentially associated with fruit softening were screened using the following criteria: 0- to 10-day time points, |log2(FPKM_ABA/FPKM_CK)| > 1, and FPKM_ABA >10. A total of 23 candidate transcription factors were identified under these conditions (Fig. 4F). Further screening of structural genes involved in ethylene biosynthesis within this module indicated that *DkACS1* (EVM0003637) expression was induced by ABA treatment (Fig. S7).

### Regulatory effect of ABA-induced *DkNAC28* on the promoter of *DkACS1*

**Figure 5:**
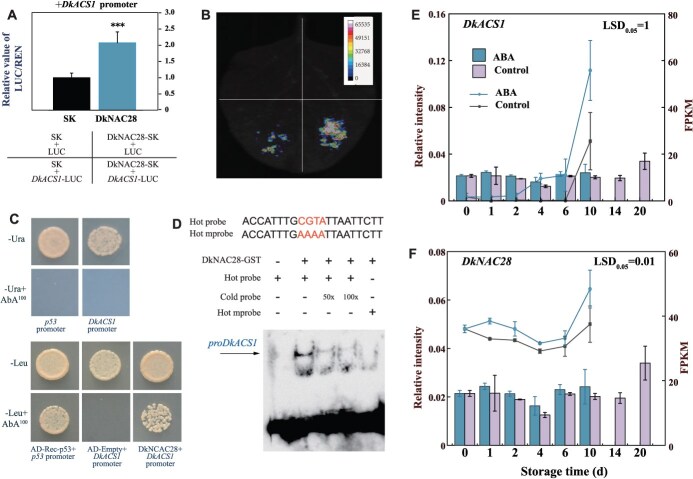
DkNAC28 enhances the promoter activity of *DkACS1* and ABA promotes the expression of *DkNAC28* and *DkACS1*. (A) Dual-luciferase assays were performed to detect the regulatory effects of DkNAC28 on the promoter of *DkACS1*. The LUC/REN fluorescence ratio obtained from the empty vector pGreenII 002962-SK (SK) plus promoter was set to 1. Values are presented as mean (±SE) of three biological replicate assays. (B) Firefly LCI in *N. benthamiana* leaves. (C) Y1H assay shows binding of DkNAC28 to the *DkACS1* promoter. The basal concentrations of aureobasidin A (AbA) used were 300 ng·ml ^− 1^. The empty vector with the *DkACS1* promoter served as negative controls and Rec-P53 and the P53 promoter were used as the positive controls. (D) EMSA showing that DkNAC28 binds to the NAC-binding of the *DkACS1* promoter. The hot probe was a biotin-labeled *DkACS1* promoter fragment (CGTA), and the cold probe was an unlabeled competitive probe (50- and 100-fold that of the hot probe). A mutant hot probe comprised a hot probe sequence with four mutated nucleotides. GST-tagged DkNAC28 was purified. The expression levels of *DkACS1* (E) and *DkNAC28* (F) were detected by RT-qPCR. The histogram represents the RT-qPCR data, and the line chart illustrates the FPKM values obtained from the transcriptome data. Values are means (±SE) from three biological replicates. The LSD_0.05_ values refer to mean comparisons between treated and untreated samples at the same time point.

The results of dual-luciferase reporter assays showed that DkNAC28 (EVM0016518) of 23 differentially expressed transcription factors enhanced the activity of the *DkACS1* promoter with fold changes of 2.1 (Fig. 5A and B, Fig. S8). The yeast one-hybrid assay was performed to further verify the interaction between DkNAC28 and the *DkACS1* promoter. The results demonstrated that DkNAC28 was capable of binding to the *DkACS1* promoter region (Fig. 5C). To further confirm this interaction, we purified the full-length DkNAC28 protein and performed an EMSA with fragments of the biotin-labeled *DkACS1* promoter containing NAC-binding site (CGTA) as the labeled probe. DkNAC28 bound directly to the NAC-binding site (Fig. 5D). RT-qPCR showed that the expression levels of *DkNAC28* and *DkACS1* in ABA-treated persimmon fruit were higher than in the control (Fig. 5E and F). These results indicate that ABA-induced *DkNAC28* enhances expression of the ethylene biosynthesis gene *DkACS1*, thereby promoting ethylene production in persimmon fruit.

### DkNAC26 and DkNAC28 functions in regulating ABA content and ethylene production in persimmon fruit

**Figure 6:**
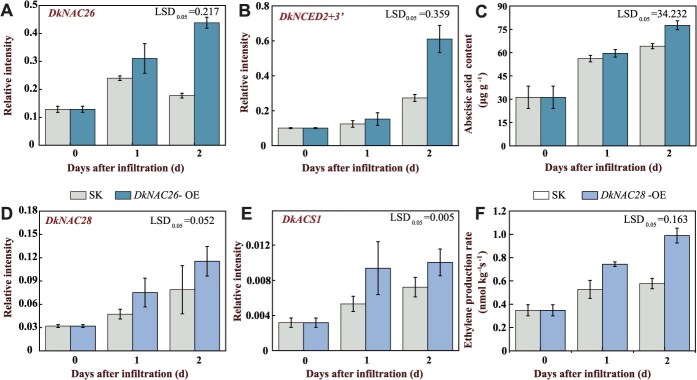
DkNAC26 and DkNAC28 respectively promote the synthesis of ABA and ethylene in persimmon fruit discs. *DkNAC26* and *DkNAC28* were overexpressed (*DkNAC26*-OE and *DkNAC28*-OE) in persimmon fruit discs. *DkNAC26* and *DkNAC28* via *A. tumefaciens* infection, with empty pGreenII 002962-SK (SK) vector serving as the control. *DkNAC26*-OE, *DkNAC28*-OE and control fruit were infiltrated, which were stored at room temperature for 2 days. (A) RT-qPCR was used to assess the expression level of *DkNAC26* in *DkNAC26*-OE persimmon fruit discs to confirm successful infection. (B) The relative expression levels of *DkNCED2 + 3′* in persimmon fruit discs was analyzed by RT-qPCR. (C) ABA content was measured. (D) RT-qPCR was used to assess the expression level of *DkNAC28* in *DkNAC28*-OE persimmon fruit discs to confirm successful infection. (B) The relative expression levels of *DkACS1* in persimmon fruit discs were analyzed by RT-qPCR. (C) Ethylene production was measured.

To further verify the effects of DkNAC26 on endogenous ABA and of DkNAC28 on endogenous ethylene, we transiently overexpressed *DkNAC26* and *DkNAC28* in persimmon fruit discs via *Agrobacterium tumefaciens* infiltration. Transient overexpression of *DkNAC26* led to higher *DkNCED2 + 3′* expression and increased ABA content compared with the control (Fig. 6A–C), suggesting that DkNAC26 promotes ABA accumulation by upregulating *DkNCED2 + 3′*, thereby accelerating fruit softening. Upon transient overexpression of *DkNAC28*, *DkACS1* transcript abundance, ACC content, and ethylene production were all higher than in the control (Fig. 6D–F, Fig. S9). These findings indicate that DkNAC28 promotes ethylene biosynthesis by inducing *DkACS1* expression, thereby facilitating fruit softening.

## Discussion

### ABA plays a crucial role in the ripening processes of both climacteric and nonclimacteric fruit

ABA is generally considered a key regulator of nonclimacteric fruit ripening. In strawberry (*Fragaria ananassa*), ABA enhances ethylene production, anthocyanin and phenolic accumulation, and phenylalanine ammonia-lyase (PAL) activity during ripening [30]. In sweet cherry, an ABA–PavARF8–PavDofs regulatory module contributes to cell wall degradation and fruit softening [31]. Moreover, ABA also regulates ripening and softening of climacteric fruits, including banana, pear (*Pyrus communis*), and tomato [32]. Exogenous ABA increases ABA content and induces expression of *LeACS2*, *LeACS4*, and *LeACO1* in tomato fruit [33]. In banana fruit, ABA induces expression of the transcription factor *MabZIP95*, which directly binds to the promoters of *MaACS7*, *MaACO2*, and *MaACO3*, thereby enhancing ethylene biosynthesis [34]. In this study, ABA reduced persimmon fruit firmness and advanced the ethylene peak (Fig. 1E–I). ABA-induced DkNAC28 activated *DkACS1* expression, promoting ACC accumulation and ethylene production in persimmon fruit (Fig. 6D–F). This regulatory mechanism accelerates the conversion of protopectin into soluble pectin, thereby leading to earlier fruit softening. The aforementioned results collectively demonstrate that ABA influences physiological traits associated with fruit quality maturation through transcriptional regulatory mechanisms, including cell wall degradation, color development, and ethylene biosynthesis. ABA plays a crucial regulatory role in the ripening processes of both climacteric and nonclimacteric fruit.

### The interactions among various plant hormones are critically involved in the regulation of fruit ripening and softening processes

**Figure 7:**
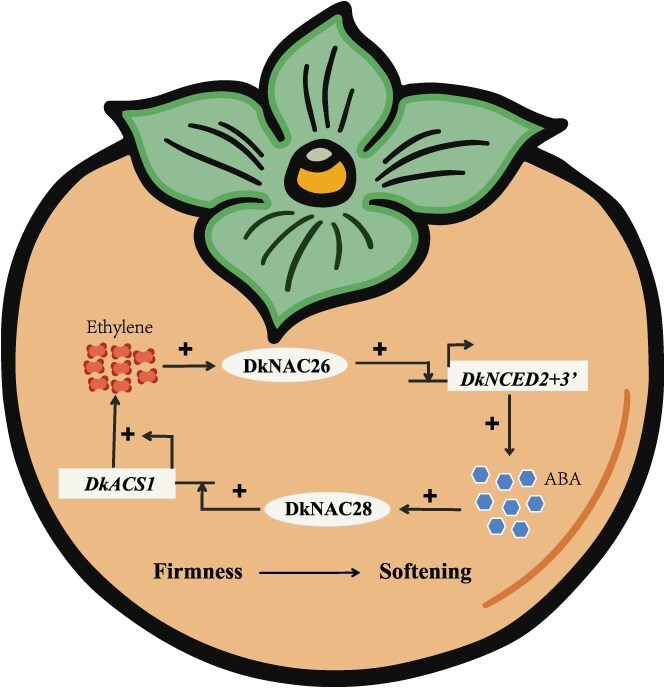
Molecular mechanism model of ethylene and ABA promoting each other and accelerating the softening of persimmon fruit. Exogenous ethylene treatment induces the expression of the transcription factor *DkNAC26*, which enhances ABA biosynthesis through binding to the promoter region of the key ABA biosynthetic gene *DkNCED2 + 3′*. Conversely, ABA treatment promotes ethylene biosynthesis by upregulating the expression of the transcription factor *DkNAC28*, which binds to the promoter of the key ethylene biosynthetic gene *DkACS1*. Ethylene and ABA exhibit a synergistic interaction during the softening process of persimmon fruit.

Ethylene is a key plant hormone that orchestrates fruit ripening and quality [35, 36]. Its promotion of softening occurs primarily through transcriptional regulation of cell wall remodeling genes. Following ethylene treatment, significant changes in ethylene-responsive transcripts were detected as early as 2 h post-treatment (Fig. 2B). In particular, the transcript abundance of the transcription factor *DkNAC26* increased markedly at 2 h relative to the control (Fig. 3G). Moreover, transcription of *DkNCED2 + 3′*, a key ABA biosynthetic gene, was rapidly upregulated at 6 h (Fig. 3F), concomitant with an increase in endogenous ABA content (Fig. 1D). Together, these observations indicate that ethylene rapidly activates downstream transcriptional cascades and modulates the biosynthesis of both ethylene and ABA, thereby accelerating ripening-associated softening. Consistent with this, in peach PpeERF2 binds the promoters of *PpeNCED2*, *PpeNCED3*, and *PpePG1* to regulate ABA biosynthesis and cell wall degradation during ripening [37]. Beyond ABA, other plant hormones including auxin, brassinosteroids, and gibberellins (GA) also regulate ethylene biosynthesis. However, the mechanisms by which ethylene integrates with these hormonal pathways remain incompletely understood [38–40]. Here, we show that ethylene induces *DkNAC26*, which in turn activates the *DkNCED2 + 3′* promoter, increasing ABA accumulation (Fig. 7). These findings provide mechanistic insight into ethylene-promoted ABA biosynthesis at the level of transcriptional regulation. Notably, exogenous ethylene does not fully reverse the GA_3_-induced delay in tomato ripening. Application of GA_3_ to ethylene-signaling mutants (Nr) or transgenic lines overexpressing *SlEBF3* still delays ripening, suggesting that GA acts in part via an ethylene-dependent pathway. GA also influences tomato ripening by suppressing ABA biosynthesis [41]. Conversely, cosilencing ABA receptors increases fruit firmness and disease susceptibility, yet ethylene can still trigger ripening in the absence of intact ABA signaling [24]. Collectively, ethylene and ABA mutually reinforce each other during ripening-associated softening, but their effects are not entirely interdependent (Fig. 7).

### The NAC transcription factor family plays a crucial role in the ripening and softening of fruit

Fruit ripening is governed by a complex network of transcription factors and regulatory components that integrate internal hormonal cues and external signals. Deciphering these regulatory mechanisms is essential for maintaining fruit quality, optimizing storage, and extending shelf life. Plant-specific NAC transcription factors are key regulators of plant growth, fruit ripening, and stress responses. In strawberry, FvNAC073 upregulates cell wall hydrolase genes *FvXTH3* and *FvPL1*, the anthocyanin regulator *FvMYB10*, and the ABA biosynthetic gene *FvNCED5*, thereby promoting fruit expansion and ripening [42]. In sweet cherry, ABA induces *PavNAC56*, which directly binds the promoters of cell wall metabolism genes (such as *PavPG2*, *PavEXPA4*, *PavPL18*, and *PavCEL8*) to activate their expression and promote softening [43]. In apple, MdNAC1-L upregulates *MdPL5*, *MdPG1*, and ethylene-related genes (*MdACS1*, *MdACO1*), thereby reducing firmness and accelerating softening [44]. In this study, *DkNAC26* and *DkNAC28* promote persimmon softening by regulating ABA and ethylene biosynthesis (Fig. 7), thereby expanding current knowledge of NAC-mediated softening, particularly through modulation of ABA biosynthesis. Collectively, these studies underscore the pivotal role of NAC transcription factors in orchestrating the molecular programs that drive fruit ripening and softening.

## Conclusion

In conclusion, we propose a molecular model that elucidates the mutual regulatory relationship between ethylene and ABA. Ethylene activates *DkNCED2 + 3′* via the transcription factor DkNAC26, thereby promoting ABA biosynthesis in persimmon fruit. Conversely, ABA induces *DkNAC28*, which in turn upregulates *DkACS1* and increases ethylene production. This bidirectional regulatory loop accelerates softening in persimmon. These insights highlight *DkNAC26* and *DkNAC28* as promising targets for postharvest regulation of fruit texture.

## Materials and methods

### Plant materials and treatments

Persimmon fruit (*D. kaki* cv. Fupingjianshi) were harvested at commercial maturity (70%–80% surface yellow) from a commercial orchard in Fuping, Shaanxi, China (34°47′N, 109°2′E) in October 2021. Fruit without visible defects and with uniform maturity, size, and color were selected. Upon arrival, fruit were equilibrated at 20 ± 1°C and randomly assigned to four groups (400 fruit per group). For the ethylene experiment, fruit were exposed in sealed chambers to 100 μl l ^− 1^ ethylene gas (commercial-grade gas ethylene from special gas company of Yuanzheng in Henan Province of China, ≥99.9% purity) and sampled every 2 h for 24 h; untreated fruit served as the ethylene control. For the ABA experiment, fruit were sprayed with 300 mg·l ^− 1^ ABA (CA1010, Coolaber, China), with water-sprayed fruit as the ABA control. Sprays were applied to runoff. At each sampling point, three biological replicates were collected (12 fruit per replicate). Samples were immediately frozen in liquid nitrogen and stored at −80°C until analysis.

### Determination of fruit firmness, ethylene production, ABA contents, and pectin content

The fruit firmness and ethylene production were measured according to Han *et al* [45]. Fruit firmness was measured using a penetrometer (FT327, Effegi, Alfonsine, Italy) at two equidistant points 90 degrees apart on the equatorial region after peel removal. A total of six persimmon fruit were placed in a 9.17-l sealed container and allowed to incubate for 1 h. For ethylene measurement, 1 ml of headspace gas was withdrawn with a sterile syringe and analyzed using a GC-14A gas chromatograph (Shimadzu, Kyoto, Japan). The contents of ABA and ACC were determined by High Performance Liquid Chromatography-Tandem Mass Spectrometry (HPLC-MS, QTRAP 5500; Sciex, USA) according to Wang *et al*. [46]. The levels of protopectin and soluble pectin were quantified following a previously established method [47].

### Transcriptome sequencing, RNA extraction, cDNA synthesis, and gene expression analysis

In total, 111 samples were subjected to RNA-seq (control and ethylene-treated fruit at 0, 2, 4, 6, 8, 10, 12, 14, 16, 18, 20, and 24 h and 2, 4, and 6 days; water- and ABA-treated fruit at 0, 2, 4, 6, and 10 days), each with three biological replicates, by Biomarker Technologies Corporation (Beijing, China). Total RNA was extracted following Wang *et al*. [46]. PrimeScriptTM RT Reagent Kit with gDNA Eraser (Cat. no. RR047A, Takara, Japan) was used to remove genomic DNA and synthesize cDNA. Gene expression was measured by RT-qPCR using the conditions described in Xu *et al* [48]. Total RNA was extracted from persimmon using a modified CTAB method. cDNA was synthesized using the TaKaRa reverse transcription kit (Code No. RR047A, Takara, Japan). Specific primers for the target genes were designed based on the Prime 3 (V. 0.4.0; http://bioinfo.ut.ee/primer3-0.4.0/), and primer specificity was confirmed by agarose gel electrophoresis. RT-qPCR was performed with an iCycler iQ5 (Bio-Rad, Hercules, CA) using a SYBR PrimeScript RT-PCR kit II (TaKaRa, Kyoto, Japan). Gene expression levels were calculated using the 2^-ΔCt method, with each experiment performed in triplicate biological repeats. All primers used for gene expression analysis are listed in Supplementary Table S1.

### Dual luciferase assay

Full-length TF coding sequences were cloned into pGreenII 002962-SK through Sac І and Sal I, and the promoters of *DkNCED2 + 3′* and *DkACS1* were inserted into pGreenII 0800-LUC by Kpn I and Nco I. *A. tumefaciens*-mediated coinfiltration was performed in *Nicotiana benthamiana* leaves following Zhu *et al* [49]. Leaves were sampled 3 days postinfiltration, and firefly luciferase activity (LUC) was normalized to Renilla luciferase (REN) using a Spark multimode plate reader (Tecan, Switzerland).

### Yeast one-hybrid assay

Coding sequences of *DkNAC26* and *DkNAC28* were cloned into pGADT7 (Clontech, USA) through Nde I and EcoR I. Promoter fragments of *DkNCED2 + 3′* and *DkACS1* were cloned into pAbAi (Clontech, USA) by Kpn I and Sal I. Yeast one-hybrid (Y1H) assays were performed as described previously [48].

### EMSA

The CDS of *DkNAC26* and *DkNAC28* were cloned into pGEX4T-1 (TaKaRa, Japan) through EcoR I and Sal I. It was then transformed into BL21 (DE3) cells to produce DkNAC26-GST and DkNAC28-GST. The DkNAC26-GST and DkNAC28-GST protein were purified as previously described [48]. Oligonucleotide probes containing NAC-binding sites (CGTA and CGTG) were synthesized and labeled with biotin (Sangon Biotech). An EMSA was performed using a Lightshift Chemiluminescent EMSA kit (Beyotime Biotechnology) according to the manufacturer’s instructions.

### *Agrobacterium* infiltration

*A. tumefaciens*-mediated transient transformation of persimmon fruit (*D. kaki* cv. Fupingjianshi) slices was employed to validate the functional roles of *DkNAC26* and *DkNAC28.* The experimental procedure was adapted from Zhu *et al* [50]. Persimmon fruit flesh was processed into circular slices with a diameter of 1 cm and a thickness of ~0.5 cm using a biopsy punch, followed by immersion in a 5% mannitol solution to maintain osmotic balance. Subsequently, the pGreenII 002962-SK vector carrying the full-length sequences of *DkNAC26* and *DkNAC28*, as well as the empty pGreenII 002962-SK vector, were individually introduced into *A. tumefaciens* cells. The bacterial suspension was adjusted to an OD600 of 0.75 and contained 10 mM MES, 10 mM MgCl_2_, and 150 μM acetosyringone. The prepared fruit slices were submerged in the bacterial suspension and incubated at 28°C for 1 h under static conditions. All procedures were conducted under aseptic conditions. Following inoculation, excess bacterial suspension was removed using sterile filter paper, and the infected slices were evenly distributed into dishes containing MS Murashige and Skoog solid medium. The dishes were sealed and transferred to a 25°C growth chamber for an additional 2 days of incubation. Sampling was conducted daily throughout the incubation period, with three biological replicates collected at each time point.

### Weighted gene coexpression network analysis

The Pearson correlation coefficients were calculated to integrate the transcriptomic and physiological data. The WGCNA package in R (Langfelder and Horvath, 2008) was applied and genes with expression values (FPKM) in any variable ≥1 and differentially expressed metabolites were selected for WGCNA. The coexpression networks were visualized with Cytoscape (v.3.9.1) [51]. Genes with significant differences in expression (i.e., log2 foldchange >1 and adjusted *P*-value <0.01) were considered DEGs and were annotated with gene ontology (GO) terms. The reference gene sequence information can be accessed through the official database website: http://www.kakiwi.zju.edu.cn/cgi-bin/persimmon/index.cgi.

### Statistical analysis

Multiple group comparisons were performed using one-way ANOVA followed by Fisher LSD *post hoc* testing, and pairwise comparisons were conducted using Student’s *t-* test. Statistical analyses were implemented in DPS 2.05 software (Zhejiang University). Graphical illustrations were generated using Origin 8.0 (Microcal Software) and Adobe Photoshop CS6. The data were tested for normality and homogeneity of variances before performing the ANOVA and *post hoc* analysis. Data are presented as mean ± SD, and statistical significance was set at *P* < 0.05 (Supplementary Table S2-5).

## Supplementary Material

Web_Material_uhag055

## Data Availability

Transcriptome data during the study were deposited in the NCBI under accession number PRJNA1392662 (the transcriptomic analysis of persimmon fruit treated with ethylene and control) and PRJNA1280690(the transcriptomic analysis of persimmon fruit treated with ABA and control).
