# Supplementary material for: ABA–ethylene crosstalk accelerates persimmon fruit softening via induction of DkNAC26 and DkNAC28
Source: Hortic Res. 2026 Feb 28;13(6):uhag055. doi: 10.1093/hr/uhag055 (PMC13242957; doi:10.1093/hr/uhag055)
Supplement: Web_Material_uhag055 [file web_material_uhag055.zip › clean Supplementary Figure legends.docx]

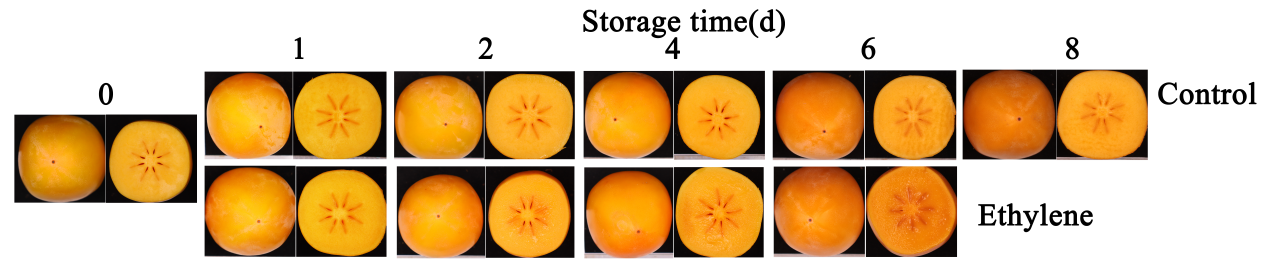


Supplemental Figure 1. Exogenous ethylene promoted softening in persimmon fruit.

Persimmon fruit was harvested and treated with ethylene. Color of the fruit flesh changes during fruit storage for 8 days.


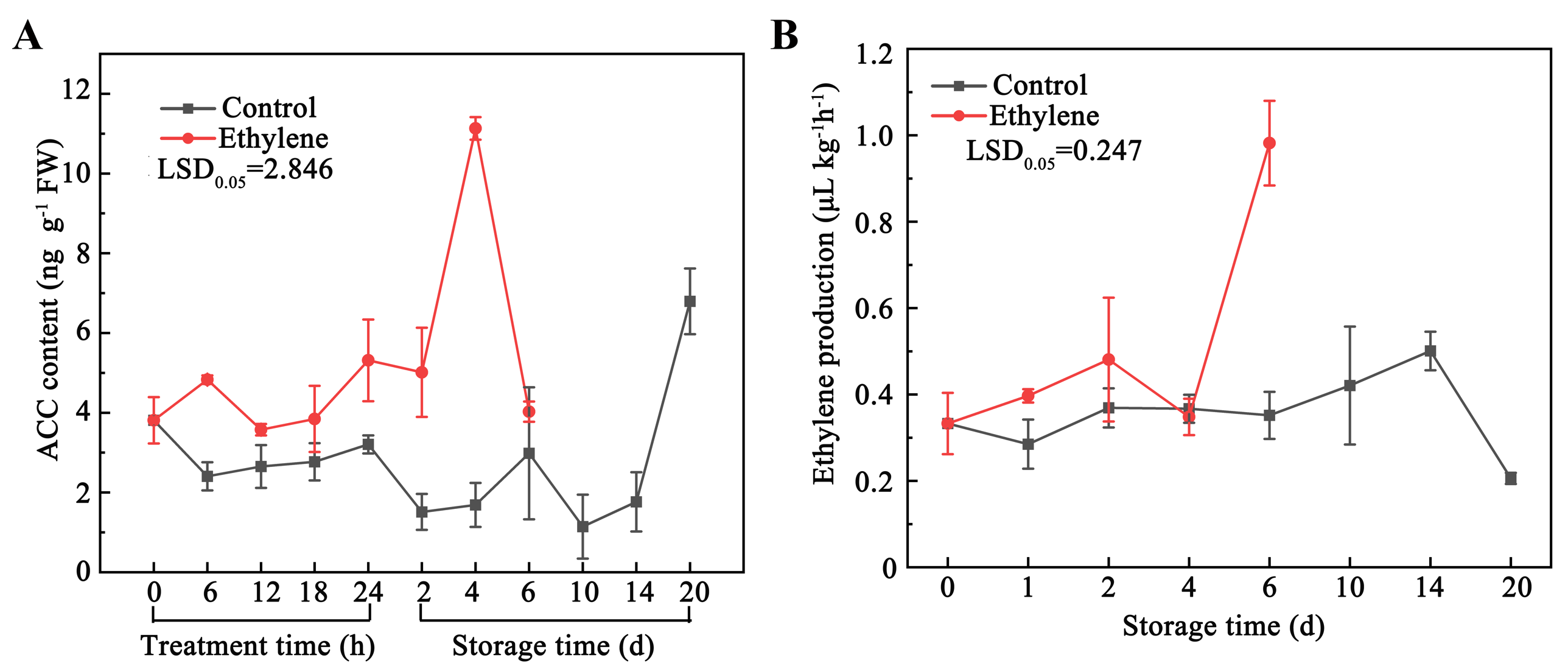


Supplemental Figure 2. Exogenous ethylene treatment promoted ethylene production in persimmon fruit. ACC content (A) and endogenous ethylene production (B) were measured after ethylene treatment. Values are the means (±SE) from three biological replicates. The LSD₀.₀₅ values refer to mean comparisons between treated and untreated samples at the same time point.


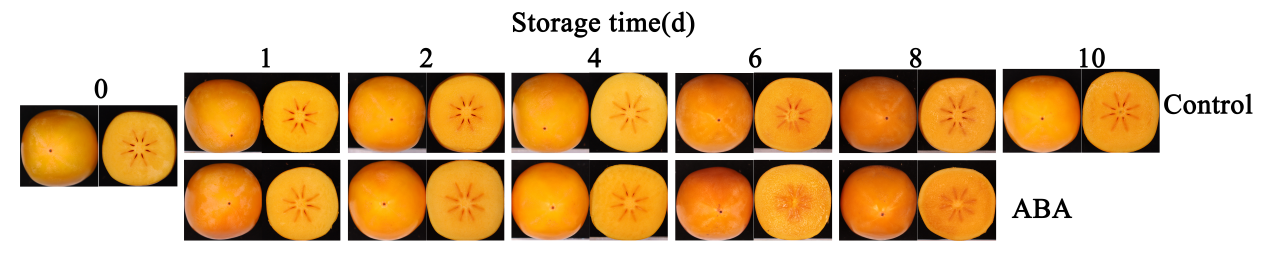
Supplemental Figure 3. ABA treatment promoted softening in persimmon fruit.

Persimmon fruit was harvested and treated with ABA. Color of the fruit flesh changes during fruit storage for 10 days.


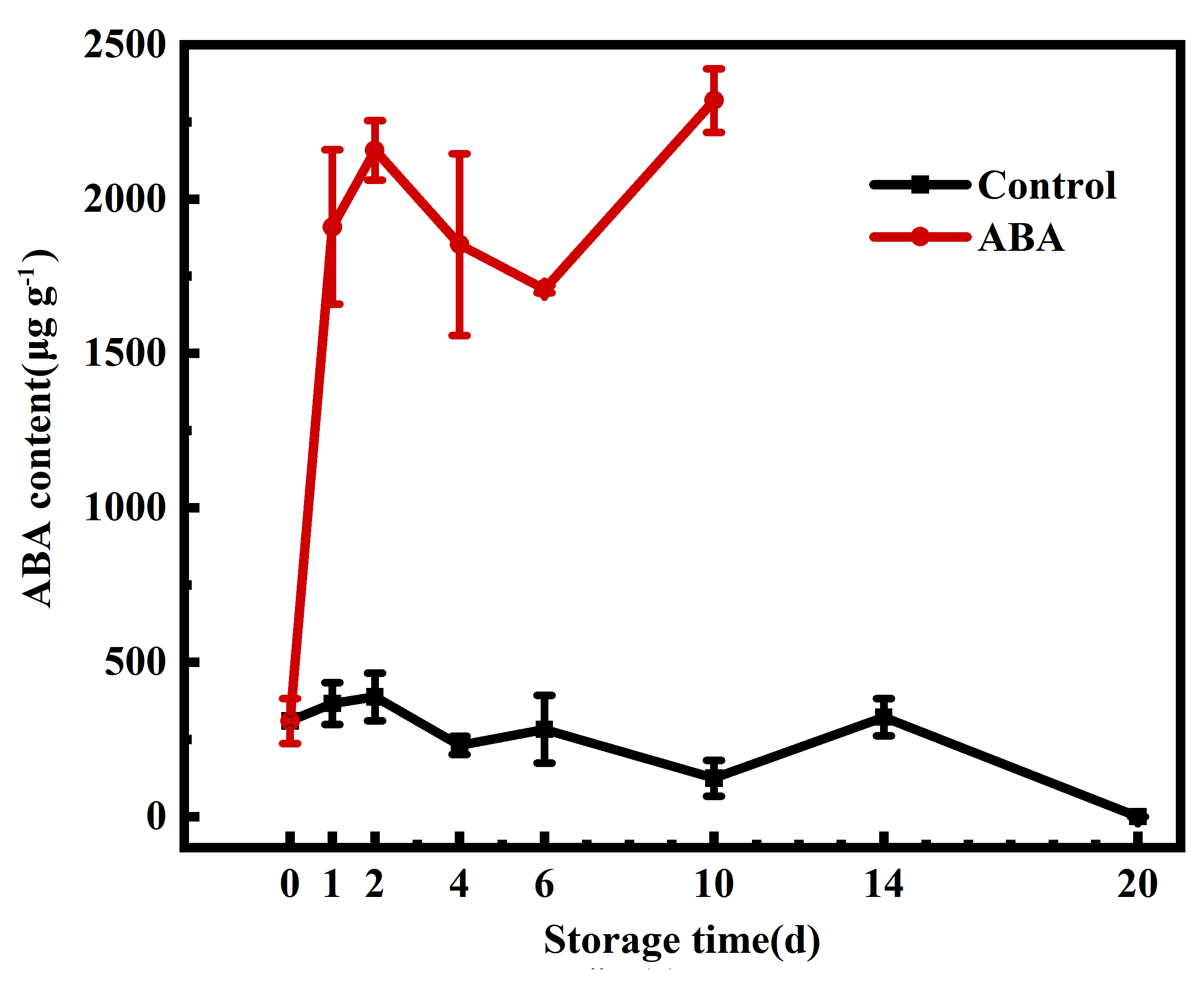


Supplemental Figure 4. ABA treatment promoted ABA content of persimmon fruit. Values are the means (±SE) from three biological replicates.


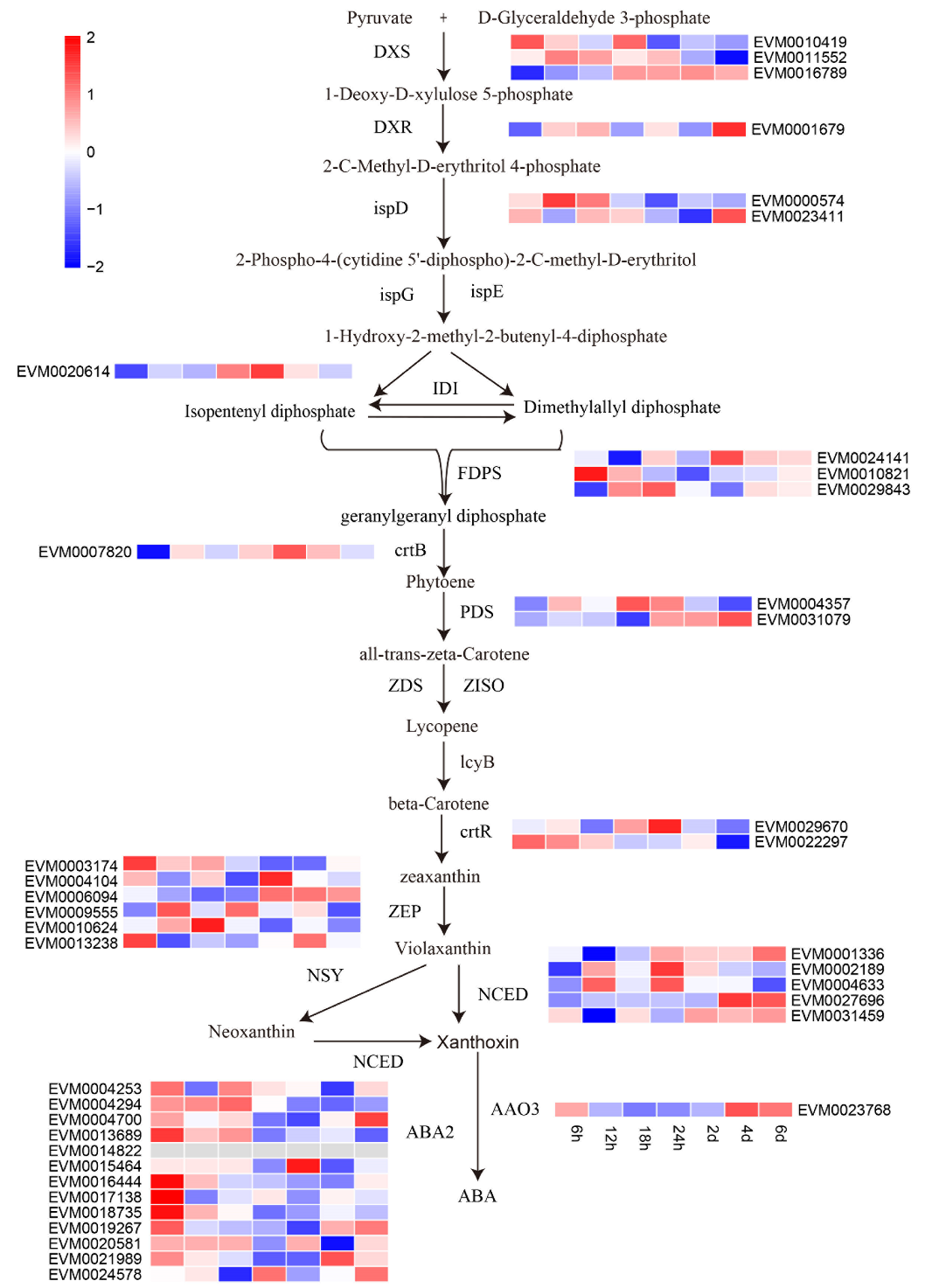


Supplemental Figure 5. Heatmap analysis of the differential expression patterns of key genes involved in the ABA biosynthesis pathway. DXR：1-deoxy-D-xylulose-5-phosphate synthase；DXR：1-deoxy-D-xylulose-5-phosphate red-uctoisomerase；ispD：2-C-methyl-D-erythritol 4-phosphate cytidylyltransferas；ispG：(E)-4-hydrox-y-3-methylbut-2-enyl-diphosphate synthase；ispE：4-diphosphocytidyl-2-C-methyl-D-eryth-ritol kinase；IDI：isopentenyl-diphosphate Delta-isomerase；FDPS：farnesyl diphosphate synthase；crtB：15-cis-phytoene synthase；PDS：phytoene desaturase；ZDS：zeta-carotene desaturase；lcyB：lycopene be-tacyclase；crtZ：beta-carotene 3-hydroxylase；ZEP：zeaxanthin epoxidase；NSY：neoxanthin synt-hase；NCED：9-cis-epoxycarotenoid dioxygenase；AAO3：abscisic-aldehyde oxidase；ABA2：xa-nthoxin dehydrogenase.


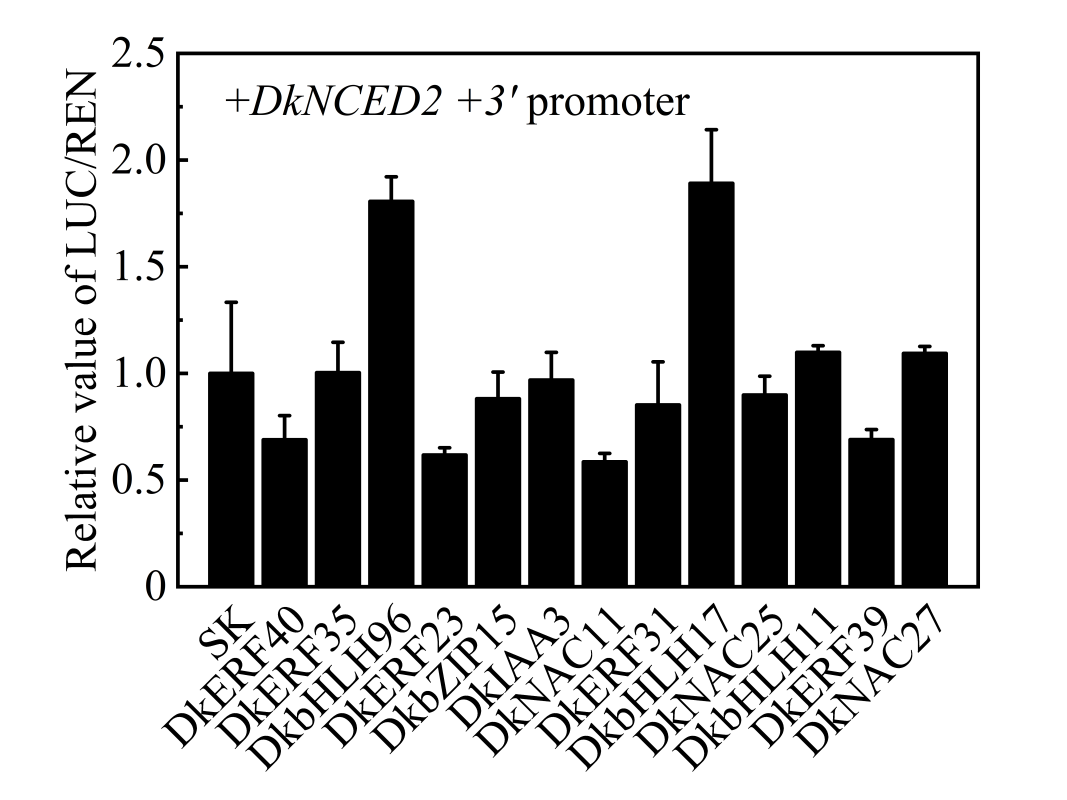


Supplemental Figure 6. The regulatory effects of 13 differentially expressed transcription factors on *DkNCED2+3’* promoter. Dual-luciferase assays were performed to detect the regulatory effects of 13 differentially expressed transcription factors on the promoter of *DkNCED2+3’* . The LUC/REN fluorescence ratio obtained from the empty vector pGreenII 002962-SK (SK) plus promoter was set to 1. Values are presented as mean (±SE) of three biological replicate assays.


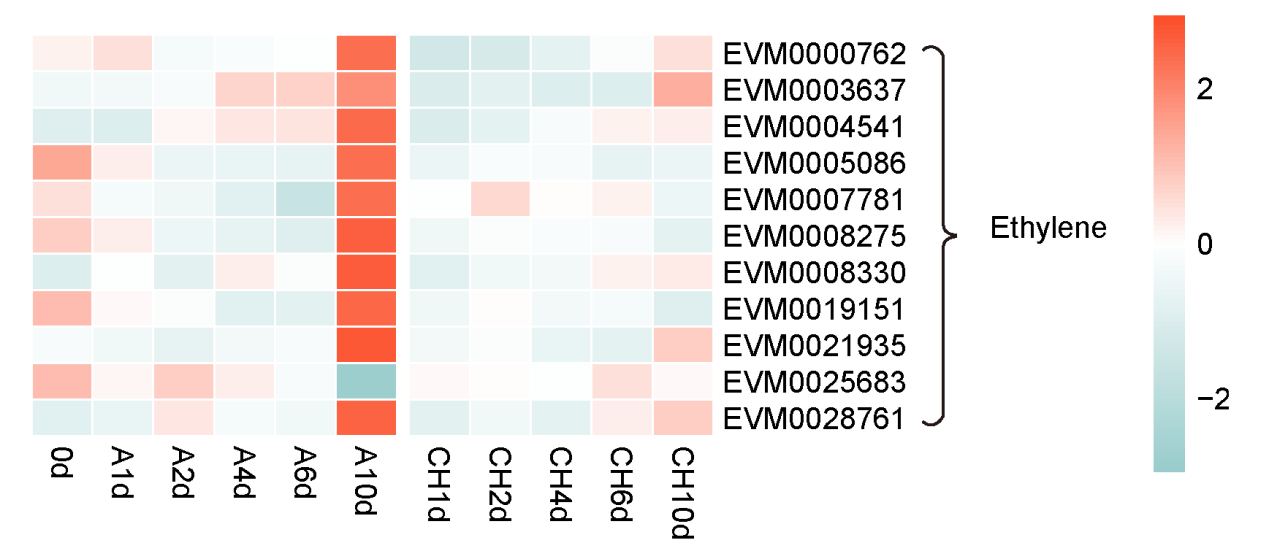


Supplemental Figure 7. Heatmap of ethylene biosynthesis genes with differential expression between control (CH) and ABA-treated (A) fruit from the RNA-seq data.


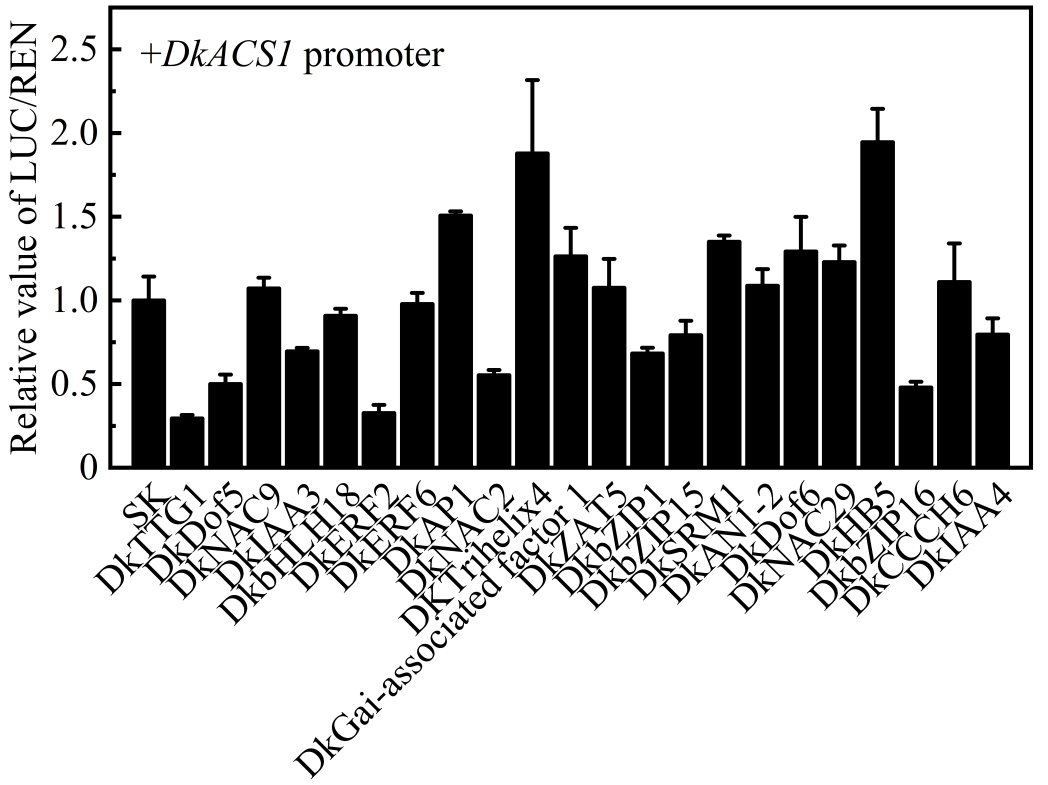


Supplemental Figure 8. The regulatory effects of 22 differentially expressed transcription factors on *DkACS1* promoter. Dual-luciferase assays were performed to detect the regulatory effects of 22 differentially expressed transcription factors on the promoter of *DkACS1* following ABA treatment. The LUC/REN fluorescence ratio obtained from the empty vector pGreenII 002962-SK (SK) plus promoter was set to 1. Values are presented as mean (±SE) of three biological replicate assays.


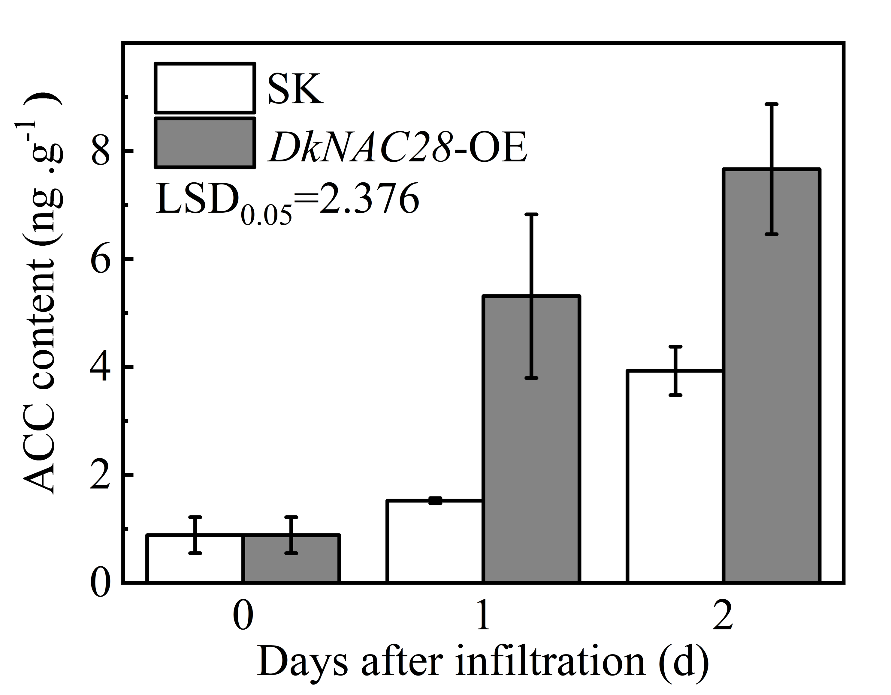


Supplemental Figure 9. ACC content was measured in *DkNAC28*-OE and control persimmon fruit discs.
